# Radiological response assessment after stereotactic body radiotherapy for spine metastases using magnetic resonance imaging: a systematic review

**DOI:** 10.1016/j.phro.2025.100840

**Published:** 2025-09-23

**Authors:** Keivan Daneshvar, Mohammadamin Shahrbaf, Johannes Heverhagen, Katarina Bryjova, Daniel M. Aebersold, Pejman Jabehdar Maralani, Arjun Sahgal, Matthias Guckenberger, Hossein Hemmatazad

**Affiliations:** a Department of Diagnostic, Interventional and Pediatric Radiology (DIPR), Inselspital, Bern University Hospital, University of Bern, Switzerland; b Department of General, Visceral and Transplantation Surgery, Heidelberg University Hospital, Heidelberg, Germany; c Department of Radiation Oncology, Inselspital, Bern University Hospital and University of Bern, Switzerland; d Department of Medical Imaging, Sunnybrook Health Sciences Centre, University of Toronto, Toronto, ON M4N 3M5, Canada; e Department of Radiation Oncology, Sunnybrook Research Institute, Toronto, ON M4N 3M5, Canada; f Department of Radiation Oncology, University Hospital Zurich, University of Zurich, Zurich, Switzerland

**Keywords:** Stereotactic body radiotherapy, Magnetic resonance imaging, Spinal metastases, Response assessment

## Abstract

**Background and purpose:**

Magnetic resonance imaging (MRI) plays a central role in evaluating treatment response after stereotactic body radiotherapy (SBRT) for spinal metastases. However, current guidelines focus mainly on conventional MRI sequences and lack standardized, comprehensive criteria for post-treatment assessment. This systematic review aimed to summarize available evidence on MRI-based response assessment following spine SBRT, emphasizing the potential of advanced MRI techniques and computational tools to improve clinical decision-making.

**Materials and methods:**

We systematically searched PubMed, Scopus, Web of Science, and Embase from their inception to August 1, 2024. Two reviewers independently screened studies on MRI-based response assessment after SBRT for spinal metastases, evaluated eligibility, and extracted data on MRI techniques, response criteria, imaging biomarkers, and clinical outcomes.

**Results:**

Thirteen studies met the inclusion criteria. Tumor volume changes assessed by sagittal T1-weighted MRI, with a minimum detectable difference of approximately 11 %, were essential for evaluating local control. T2 signal alterations and reductions in dynamic contrast-enhanced (DCE) MRI perfusion parameters, such as K_trans_ and V_p_, correlated with improved outcomes, including pain relief and local control. Pseudo-progression and intralesional fatty content were identified as key imaging features that may mimic progression. Diffusion-weighted imaging (DWI) and apparent diffusion coefficient (ADC) mapping showed promise as response biomarkers, but lack clinical validation. Radiomics and machine learning models improved predictive accuracy for treatment outcomes and individual follow-up strategies.

**Conclusions:**

MRI provides essential morphological and functional biomarkers for response assessment after spine SBRT. Standardized, multi-parametric MRI protocols and computational tools are needed to optimize patient care.

## Highlights

• MRI is essential for response assessment after SBRT for spinal metastases.
• T2 signal, tumor volume, and DCE-MRI perfusion are key response biomarkers.
• Pseudo-progression and fatty marrow changes complicate MRI interpretation.
• Radiomics and machine learning improve MRI-based outcome prediction.
• Standardized, multi-parametric MRI protocols are needed for reliable follow-up.

## Graphical abstract

**Figure:**
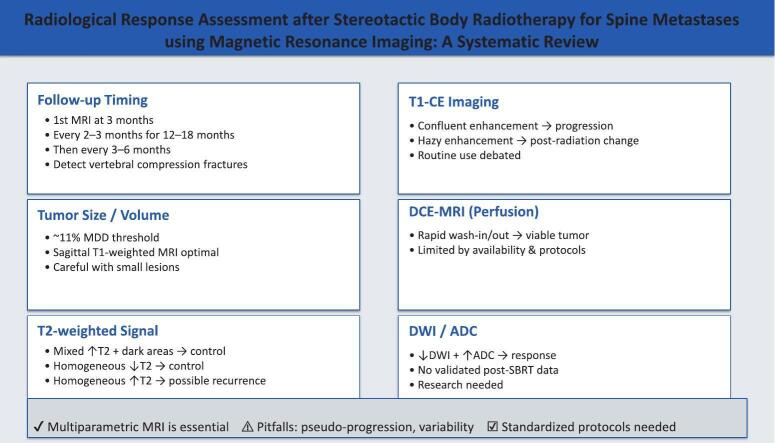

## 1 Introduction

Metastatic disease most commonly affects the bones, with over two-thirds of cancer patients developing spinal metastases [1]. If left untreated, spinal metastases can affect quality of life through pain, fractures, spinal instability, and neurological deficits due to malignant spinal cord compression (MSCC) [2]. Stereotactic body radiotherapy (SBRT) is an effective treatment for spinal metastases, delivering high radiation doses while sparing adjacent normal tissue. SBRT shows promising outcomes in local tumor control, pain relief, and preservation of neurological function [3]. However, accurately assessing treatment response after SBRT remains challenging in clinical practice.

**Fig. 1:**
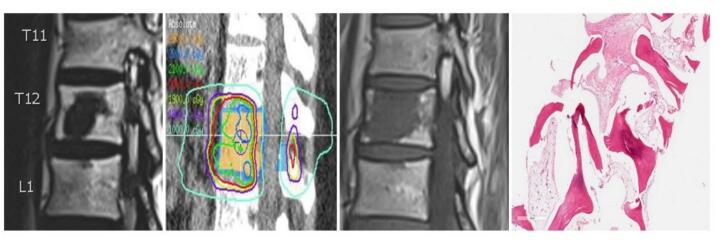
Patient treated with 24 Gy in 2 fractions with ER/PR+ oligo-metastatic breast cancer. 10 years later while on no endocrine therapy a follow-up MRI showed this sudden T1 signal change within the treated vertebral body. The biopsy showed fibroses and no active tumor.

Magnetic resonance imaging (MRI) is a key modality for monitoring response to SBRT in patients with spinal metastases [4]. Compared to conventional imaging, MRI provides superior soft tissue contrast, enabling detailed assessment of tumor characteristics such as volume changes, signal intensity variations, and vascular properties [5]. MRI also detects subtle morphological and micro-environmental changes, which is essential for distinguishing true progression from pseudo-progression (PP) and other treatment-related effects like necrosis or fibrosis (Fig. 1) [6]. Osseous PP is more common in lytic tumors and varies with the primary cancer type, making its recognition on MRI critical to avoid misinterpreting PP as disease progression [7].

Current guidelines primarily rely on endpoints such as pain relief, neurological status and survival to evaluate SBRT effectiveness. While essential, these measures may not capture the complexity of tumor response, especially when imaging changes precede clinical symptoms. This systematic review aims to evaluate the role of MRI in improving response assessment for spinal metastases after SBRT.

## 2 Materials and methods

### 2.1 Study design

This systematic review evaluated MRI for response assessment after SBRT for spinal metastases, following PRISMA guidelines [8].

### 2.2 Search strategy

We conducted a comprehensive literature search in PubMed, Web of Science, Scopus, and Embase with no start date restriction, covering publications up to August 1, 2024, to identify relevant studies. To enhance the thoroughness of the search, we also screened the reference lists of all included articles for additional publications. We placed no restrictions on publication date or language to ensure broad coverage. The search strategy included the following keywords: (“stereotactic body radiotherapy” OR “SBRT” OR “stereotactic ablative radiotherapy” OR “SABR” OR “stereotactic radiation therapy” OR “stereotactic radiotherapy”) AND (“spinal metastasis” OR “spinal metastases” OR “spine metastasis” OR “spine metastases” OR “vertebral metastasis” OR “vertebral metastases” OR “metastatic spinal tumors” OR “metastatic vertebral tumors” OR “secondary spinal tumors” OR “secondary vertebral tumors”) AND (“magnetic resonance imaging” OR “magnetic resonance tomography” OR “MRI” OR “MRT”). Eligibility criteria

Eligibility followed the PICO framework: patients with spinal metastases treated with SBRT; MRI-based response assessment; no comparator; outcomes focused on MRI response criteria. Included studies used MRI to assess SBRT response. Excluded were non-human studies, abstracts, reviews, case reports (<10 patients), or studies without MRI as the primary assessment.

### 2.3 Data extraction and evidence synthesis

Two reviewers independently screened titles and abstracts, removed duplicates, and assessed full texts for eligibility. Data extraction focused on MRI techniques, response criteria, outcomes, and correlations with clinical findings. MRI-based variables for evaluating response to SBRT in spinal metastases were thoroughly analyzed.

## 3 Results

### 3.1 Summary of screening

**Fig. 2:**
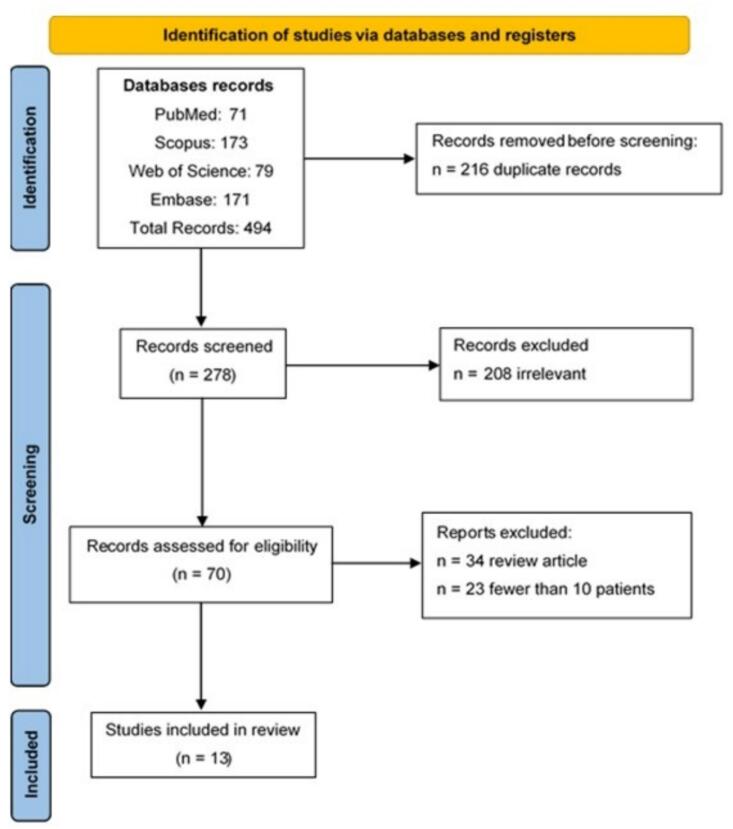
PRISMA flowchart of the study.

We identified 494 records through database searches and removed 216 duplicates. We screened 278 articles by title and abstract, excluding 208 due to irrelevance, 34 as review articles, and 23 with fewer than 10 patients. We included 13 studies in the evidence synthesis [6,9,[10], [11], [12], [13], [14], [15], [16], [17], [18], [19], [20]]. Fig. 2 illustrates the PRISMA flowchart of the study selection process.

Table 1 provides an overview of the 13 included studies, detailing sample size, fractionation schemes, evaluated parameters, and key findings.

**Table 1:** Overview of characteristics and key findings from the 13 studies included in the systematic review.

**Study**	**Sample size (patients)**	**Fractionation**	**Evaluated Parameters**	**Results**
Amini et al. 2016 [6]	37	Single-fraction (24 Gy, 18 Gy, 16 Gy) or multiple fractions (27 Gy in 3 fractions, 30 Gy in 5 fractions)	Osseous pseudo-progression (OPP)	5 lesions (14 %) showed osseous pseudo-progression
Hwang et al. 2012 [9]	44	35.3 ± 8.4 Gy in 1–6 fractions	Volume and signal intensity changes	32 lesions (40.5 %) decreased in volume, 39 (49.4 %) remained unchanged, 8 (10.1 %) increased. T2 signal intensity increased in 89.9 % of lesions, contrast enhancement decreased in most lesions, with persistent enhancement in only 4 lesions
Chen et al. 2023 [18]	194	18–24 Gy/1 fraction, 24–30 Gy/3 fractions, 30–40 Gy/5 fractions	Radiomics models performance from T1WI, T2WI, FS-T2WI sequences, combined model with clinical features	The combined model achieved the best performance with an AUC = 0.828. The optimal model based on MRI sequences alone achieved an AUC of 0.825
Chen et al. 2023 [19]	89	18–24 Gy/1 fraction, 24–30 Gy/3 fractions, 30–40 Gy/5 fractions	DCE-MRI parameters (K^trans^)	K^trans^ (tumor perfusion) was an independent predictor for pain relief
Chen et al. 2024 [20]	446	24 Gy in 2 fractions, 28 Gy in 2 fractions	MRI follow-up intervals, risk stratification for spine progression	Risk stratification model divided patients into low, intermediate, and high-risk groups. Spine progression rate at 4yr varied from 23.4 % (low-risk) to 51.8 % (high-risk) groups
Spratt et al. 2016 [10]	9	24 Gy in 1 fraction, 27 Gy in 3 fractions, 30 Gy in 3 fractions	Perfusion parameters (K^trans^, V_p_) pre- and post-SBRT, correlation with local control	K^trans^ and V_p_ significantly decreased post-SBRT. K^trans^ accurately predicted local failure in all cases. Combination of K^trans^ and V_p_ showed 100 % accuracy in predicting local control
Tseng et al. 2018 [11]	145	24 Gy in 2 daily fractions	Tumor response criteria as defined by SPINO group	MRI-based outcomes: high rates of local control (82 % at 2yr) and low rates of VCF (13.8 % at 2yr)
Zeng et al. 2019 [12]	52	Cervical spine: 24–35 Gy (1–5 fractions)Sacral spine: 24–30 Gy(2–5 fractions)	Tumor response criteria as defined by SPINO group	MRI-based outcomes: higher local control (93 % vs 79 %) and lower VCF rates (0 % vs 5.4 %) in cervical than sacral segments
Chen et al. 2021 [13]	27	25–40 Gy (3–5 fractions)	Perfusion parameters (K^trans^, K_ep_, V_e_), pre- and post-treatment changes.	Post-treatment K^trans^ and K_ep_ were significantly lower in the non-progression disease group. Post-treatment V_e_ was significantly higher in the non- non-progression disease group indicating better response
Jabehdar Maralani et al. 2021 [14]	142	24 Gy in 2 fractions	Volume changes in metastasis after SBRT	Given a MDD of 10.9 %, for small GTVs, larger (>37 %) changes were required before local failure could be determined, compared to 11 % to 13 % for medium/large tumors
Sahgal et al. 2021 [15]	229	24 Gy in 2 fractions versus 20 Gy in 5 fractions	Tumor response criteria as defined by SPINO group	Routine MRIs used for follow-up. At 3 months, complete pain response was achieved in 35 % of SBRT patients versus 14 % with conventional radiotherapy; at 6 months, the rates were 32 % vs. 16 %, respectively.
Correia et al. 2022 [16]	35	24–42 Gy (2–7 fractions)	Volume and signal intensity changes	Increased volume was observed in 20 % of patients, while 68.6 % showed stable disease. Signal intensity changes (T2-SI) included homogeneous bright, dark spots, totally dark signal, and intermediary changes, with progression often indicating significant signal intensity alterations
Vellayappan et al. 2022 [17]	12	24–27 Gy (3 fractions) 24 Gy (2 fractions)	Perfusion parameters (K^trans^, K_ep_, V_e_), pre- and post-treatment changes.	K^trans^ showed a significant reduction post-SBRT. V_p_ and V_e_ showed initial increase at 1-week post-SBRT before decreasing below baseline by 12 weeks

### 3.2 Tumor volume changes

MRI-based tumor volume changes are crucial for evaluating SBRT efficacy in spinal metastases [21]. Stable or decreased volume typically indicates local control. A key reference is the SPINO-group consensus, summarizing expert practices for assessing tumor response. Local progression is defined as a clear increase in tumor volume or linear dimension, new or progressive epidural disease, or neurological decline linked to epidural disease with corresponding MRI changes. Equivocal cases like osseous pseudo-progression require follow-up imaging to confirm progression, with failure dates backdated if confirmed [22].

A retrospective study used T1- and T2-weighted MRI to assess tumor volume changes after SBRT (mean 35.3 ± 8.4 Gy, 1–6 fractions) in 44 patients with 79 lesions [9]. Tumor volumes showed 40.5 % decreased, 49.4 % unchanged, and 10.1 % increased. Most lesions had good local control, but only pre- and post-treatment MRIs were compared, limiting dynamic change assessment [9].

Correia et al. [16] evaluated response after SBRT for spinal and non-spinal metastases using T1- (native and contrast) and T2-weighted MRI. Median dose was 24 Gy (24–42 Gy) over 2–7 fractions. Tumors showed 11.4 % partial response, 68.6 % stable disease, and 20 % progression. Local control was 80 %, but 57.1 % died despite 70 % maintaining control. Lack of size cut-offs and irregular MRI follow-ups limited dynamic change assessment.

Tseng et al. reported outcomes after SBRT (24 Gy in 2 fractions) for spinal metastases using T1- and T2-weighted MRI per SPINO guidelines. Tumors were classified by volume change after 15 months [11].

Zeng et al. [12] studied cervical and sacral metastases (median 24 Gy in 2 fractions) with MRI every 2–3 months. Anatomical complexity hindered MRI assessment. Two-year local control was 92.7 % (cervical) and 78.7 % (sacral), but dynamic changes were not reported.

Jabehdar Maralani et al. developed a quantitative MRI framework for assessing SBRT response. In 142 spinal segments with at least two MRIs (3 and 6 months post-SBRT), a minimum detectable difference (MDD) of 10.9 % in gross tumor volume (GTV) defined meaningful change. Smaller tumors required larger thresholds (37.2 %–71.3 %) to detect progression. GTV change between baseline and 6 months best predicted 1-year progression-free survival [14]. A related study confirmed that >3 mm lesion growth or a 1.67-fold area increase strongly indicated progression, with >89 % sensitivity and specificity [23].

In conclusion, MRI-based tumor volume assessment provides essential insights after SBRT for spinal metastases. Stable or reduced volumes suggest favorable outcomes, but pseudo-progression and inconsistent thresholds highlight the need for standardized, serial imaging.

### 3.3 T2 signal intensity and contrast enhancement

Alterations in T2 signal intensity and T1-contrast enhancement on MRI are essential for assessing spinal metastases response to SBRT. These MRI parameters provide insights into tumor physiology, treatment efficacy, and help differentiate treatment-related changes from progressive disease [24]. In the study by Hwang et al. response assessment after spinal SBRT partly relied on T2 signal intensity changes, categorized into four types: (1) no change, (2) homogeneous increase, (3) increase intermixed with dark intensity and (4) complete darkening [9]. The most common response, seen in 86.1 % of lesions, was increased T2 signal intermixed with dark areas, typically associated with stable or decreased tumor volume, indicating local control. Interestingly, this pattern also showed significantly lower rates of persistent contrast enhancement. Less common patterns, such as homogeneous increase or complete darkening, also provided valuable insights, suggesting effective treatment or possible recurrence, respectively. These findings emphasize the role of T2 signal changes in post-SBRT imaging [9]. Similarly, Correia et al. applied a comparable classification for post-SBRT T2-weighted MRI [16]. They reported no change in 2.3 %, homogeneous increase in 7 %, increased intensity intermixed with dark areas in 60.5 %, and complete darkening in 30.2 % of cases. Most metastases exhibited either increased T2 signal with dark areas or complete darkening, both indicating excellent local control [16].

**Fig. 3:**
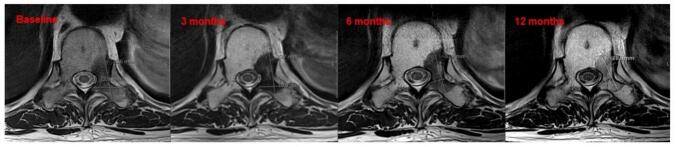
Baseline and serial T2-weighted MRI images obtained at 3, 6, and 12 months following SBRT to a T10 metastasis demonstrate marked post-treatment signal alterations.

Fig. 3 shows baseline and serial T2-weighted MRI at 3, 6, and 12 months post-SBRT in a patient with prostate cancer metastasis to T10. SBRT was delivered in three fractions of 8 Gy, prescribed to the 80 % isodose line. Signal alterations with increased intensity intermixed with low-signal areas reflect treatment response and local control, alongside visible tumor size reduction at 12 months.

These findings underline the value of T2 signal changes for accurate post-SBRT response evaluation.

### 3.4 Perfusion parameters from DCE-MRI

Dynamic contrast-enhanced (DCE)-MRI provides important insights into the perfusion characteristics of spinal metastases, offering quantitative assessment of tumor vascularization and treatment response after SBRT. Key perfusion parameters from DCE-MRI include K_trans_ (volume transfer constant), K_ep_ (rate constant), V_p_ (plasma volume), and V_e_ (extracellular extravascular space volume fraction), all of which help evaluate treatment efficacy and predict clinical outcomes [25].

A pilot study assessing spinal metastases treated with SBRT demonstrated significant reductions in K_trans_ and V_p_, with a 76 % decrease in V_p_ strongly correlating with local control. Conversely, a 30 % increase in V_p_ predicted local recurrence, with DCE-MRI detecting recurrences up to 6.6 months earlier than standard MRI [26].

In sarcoma spine metastases, DCE-MRI revealed post-SBRT reductions of up to 63.2 % in K_trans_ and V_p_, which correlated with tumor control. The K_trans_ max and a composite multi-parametric MRI (mpMRI) score demonstrated 100 % accuracy in predicting local failure, highlighting the value of these parameters as early biomarkers [10].

Chen et al. identified K_trans_ as a predictor of pain relief and overall response, showing that lower post-treatment K_trans_ values correlated with better outcomes [19]. Vellayappan et al. confirmed that reductions in V_p_ and V_e_ indicated decreased tumor perfusion and vascularity, reflecting successful treatment. These reductions were consistent over time, supporting their use for long-term monitoring. [17]. Chen et al. further observed that increased V_e_ after treatment was associated with better outcomes, as it reflected replacement of tumor tissue with fibrosis or necrosis, confirming the utility of DCE-MRI in response assessment [13].

### 3.5 Diffusion-Weighted Imaging (DWI)

DWI is an MRI technique that assesses the free movement of water molecules within tissue, providing microstructural information that complements conventional imaging, particularly for evaluating tumor cellularity. High nuclear–cytoplasmic ratios and cell density restrict fluid motion, increasing DWI signal intensity [27]. Although some studies have explored DWI for response assessment after conventional radiotherapy, its role following SBRT remains unstudied [28].

**Fig. 4:**
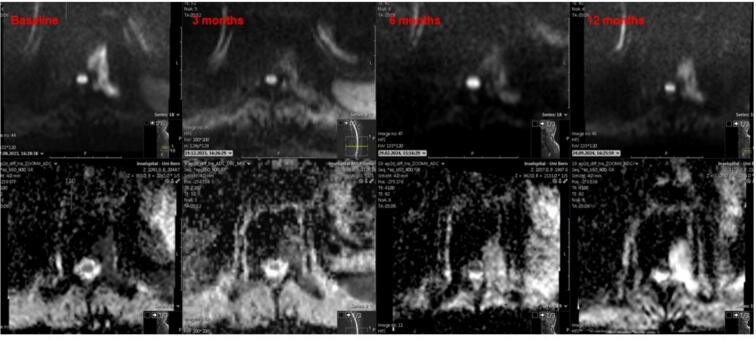
Baseline and serial DWI (upper row)/ADC (lower row) MRI images obtained at 3, 6, and 12 months following SBRT to a T10 metastasis (same patient in figure 3).

Fig. 4 presents baseline and follow-up diffusion-weighted and ADC MRI images at 3, 6, and 12 months from the same patient in Fig. 3. A clear reduction in diffusion restriction appears at 3 months post-SBRT, with corresponding ADC changes. By 12 months, a shine-through effect develops, reflecting treatment-related tissue alterations.

### 3.6 Osseous pseudo-progression and intra-lesional fatty content

Pseudo-progression (PP) is a phenomenon seen after SBRT for spinal metastases, where imaging suggests tumor progression despite effective treatment [29]. This complicates post-SBRT MRI interpretation, making accurate identification essential to avoid unnecessary interventions and properly assess treatment response. Amini et al. investigated PP prevalence and timing, finding it occurred in 14 % of lesions, particularly after single-fraction SBRT [6]. Lesions showed transient size increases and MRI signal changes between 9.7 and 24.4 weeks post-treatment, which later regressed without further intervention. The study suggested this reflects a dose-related inflammatory response rather than true progression [6].

Another study reported PP in 37 % of spinal metastases treated with SBRT, more frequently in lytic than sclerotic lesions [7]. PP onset varied by tumor type: renal cell carcinoma showed earlier and prolonged onset (106–504 days), while prostate cancer showed a later, narrower window (183–296 days). Most PP cases resolved without intervention and were confined to the vertebral body. Extending the PP assessment window beyond six months and considering tumor histology is recommended to avoid misclassifying PP as true progression [7].

No studies have yet used biopsy to distinguish PP from true progression, though such research is highly anticipated to improve diagnostic accuracy. Identifying PP relies on careful radiological interpretation and serial imaging to differentiate transient treatment-related changes from real tumor growth [30]. Amini et al. emphasized recognizing the temporal nature of PP, which peaks within the first few months post-SBRT and resolves over time [6].

Intralesional fatty content is an additional post-treatment change that can be detected on MRI in patients with spinal metastases who have undergone SBRT. Park et al. explored the appearance of intralesional fatty content in bone metastases following treatment, particularly in relation to changes observed on T1-weighted MRI and CT scans [31]. The study demonstrated that after SBRT or systemic therapy, some lesions developed post-treatment intralesional fatty content (PIFAT), which could be detected as hyper-intensity areas on T1-weighted images. This fatty content is believed to represent the replacement of necrotic tumor tissue with fatty marrow, indicative of a positive treatment response [31].

The presence of PIFAT is typically regarded as a positive finding, suggesting that the tumor has experienced substantial necrosis. However, interpreting these changes requires careful consideration, as the return of normal marrow signal intensity (indicating fatty replacement) could be mistaken for residual or recurrent disease if not properly understood. Park et al. emphasized the importance of correlating T1-weighted MRI findings with other imaging modalities, such as CT, to confirm the presence of fatty content and avoid diagnostic errors [31].

### 3.7 Vertebral compression fractures

Vertebral compression fractures (VCFs) are a known complication after SBRT for spinal metastases and can closely mimic local tumor progression on imaging, posing a diagnostic challenge. Sahgal et al. reported that VCFs may result from SBRT or occur alongside tumor failure, emphasizing the need for accurate differentiation to avoid mismanagement [32]. A review on spinal metastasis management highlighted that post-SBRT VCFs can resemble tumor regrowth, and in unclear cases, biopsy may be necessary for diagnosis [33]. A single-institution study also found that VCFs and other structural changes can be mistaken for disease progression, underscoring the need for standardized radiologic criteria and clinical correlation [16]. DCE-MRI perfusion parameters offer high diagnostic value in distinguishing malignant from benign VCFs, especially when conventional MRI is inconclusive [34]. Geith et al. [35] further demonstrated that quantitative DCE-MRI with compartmental modeling can differentiate malignant from osteoporotic fractures, reinforcing the importance of multidisciplinary evaluation and cautious interpretation of imaging after SBRT.

### 3.8 Radiomics and machine learning models

Radiomics and machine learning have advanced medical imaging, offering new tools for evaluating spinal metastases response to SBRT. Radiomics extracts quantitative features from medical images to characterize tumor biology, while machine learning enhances treatment outcome predictions.

In a study by Chen et al. [18], radiomics features from MRI scans of 194 patients treated with SBRT (18–40 Gy, 1–5 fractions) were analyzed to predict treatment outcomes. Features from T1-, T2-, and fat-suppressed T2-weighted MRI were used to build predictive models with support vector machines, random forests, and neural networks. A combined model integrating radiomics and clinical data showed superior performance, with an AUC of 0.828. Key predictors included lesion size changes, T1 hypo-intensity, and Bilsky grade, demonstrating radiomics' value in guiding SBRT decisions [18].

Another study by Chen et al. [20] developed a risk model for MRI surveillance after spine SBRT, categorizing patients as low-, intermediate-, or high-risk based on epidural disease, paraspinal disease, and SINS. The 4-year spine progression risk ranged from 23.4 % to 51.8 %, informing personalized MRI follow-up intervals.

Radiomics and machine learning thus enable non-invasive, accurate treatment response assessment and personalized follow-up, improving outcomes and resource use in SBRT-treated patients.

### 3.9 Clinical and imaging correlations

Understanding how imaging parameters relate to clinical outcomes is essential for optimizing treatment and avoiding unnecessary interventions. Pain relief remains a key clinical marker for SBRT effectiveness in spinal metastases. Studies show strong correlations between MRI findings and pain reduction. Chen et al. [19] reported that decreased K_trans_ values on DCE-MRI predicted significant pain relief after SBRT. Sahgal et al. [15] showed that SBRT offers better pain control than conventional radiotherapy, with MRI confirming superior local tumor control. T1- and T2-weighted MRI tracked tumor size and signal changes, linking stable or reduced volumes to improved outcomes. These results highlight MRI’s role in monitoring tumor response and predicting pain relief.

## 4 Discussion

This systematic review summarizes existing evidence on MRI-based response assessment after SBRT for spinal metastases. As SBRT is now standard for oligo-metastatic disease (OMD) [36], defining precise radiological criteria for local control is essential. Current guidelines, including ESTRO [37] and SPINO [22] recommendations, aid clinical practice but focus mainly on T1- and T2-weighted MRI without specific linear or volumetric cut-offs. They often overlook advanced imaging biomarkers like T2 signal alterations and DCE-MRI perfusion parameters, which provide critical insight into tumor physiology and vascular changes. A recent review emphasized MRI's key role in treatment planning and response evaluation after spine SBRT [27].

A comprehensive MRI approach using both morphological and functional sequences is essential for assessing response after spine SBRT. In the current review, MRI showed that most metastases after SBRT had stable or reduced volumes, indicating local control. T2 signal changes and reduced DCE-MRI perfusion parameters correlated with pain relief. Radiomics with machine learning improved treatment response prediction, highlighting the value of advanced MRI methods. However, no systematic data exist on dynamic changes in serial follow-up MRIs, leaving the optimal timing for assessment unclear.

Here, we summarize the role of different MRI sequences in imaging based response assessment following spine SBRT (Table 2):

1) Follow-up timing
  • First MRI at 3 months, then every 2–3 months for 12–18 months, followed by every 3–6 months.
  • Essential for detecting vertebral compression fractures (VCFs) and guiding management.
2) Tumor size/volume
  • Use a ∼11 % minimum detectable difference (MDD) to reliably identify volume changes.
  • Sagittal T1-weighted MRI is optimal; careful interpretation needed, especially for small lesions.
3) T2-weighted signal patterns
  • Mixed increased T2 signal with dark areas or homogeneous T2 signal decrease indicates local control.
  • No change or homogeneous T2 signal increase may signal recurrence, requiring close follow-up.
4) T1-weighted contrast-enhanced images
  • Routine use is debated.
  • Confluent hypo-intensity with confluent enhancement suggests progression.
  • Hazy hypo-intensity with hazy enhancement likely reflects post-radiation changes.
5) DCE MRI (perfusion imaging)
  • Rapid contrast wash-in and early wash-out may indicate viable tumor.
  • Limited use due to availability and need for specialized protocols.
6) DWI/ADC mapping
  • Decreased DWI signal and increased ADC values may suggest treatment response.
  • No validated post-SBRT data; further research needed.

**Table 2:** Role of different MRI sequences in imaging based response assessment following spine SBRT.

Parameter	Points to consider	Remarks
Follow-up Timing	First MRI at 3 months, then every 2–3 months for the first 12–18 months after SBRT, then every 3–6 months	Crucial time points for detecting vertebral compression fractures (VCFs) and guiding management
Tumor Size/Volume	10.9 % (∼11 %) minimum detectable difference (MDD) has been shown as a reliable cut off threshold for volume change detection on spine MRI	Sagittal T1-weighted imaging is optimal; careful interpretation required for small tumors
T2-Weighted Signal Patterns	Mixed increased T2 signal with dark areas or homogeneous decease in T2 signal have been shown to correlate with local control	No change or homogeneous T2 signal increase may indicate recurrence, requiring closer follow-up
T1W Contrast-Enhanced Imaging	Routine use is controversial; however, confluent hypo-intensity with confluent enhancement might suggest progression	Hazy hypo-intensity with hazy enhancement likely represents post-radiation changes
DCE-MRI (Perfusion Imaging)	Rapid contrast wash-in and early wash-out is suggestive of vital tumor tissue	Limited by availability and need for specialized protocols; not routinely performed
DWI/ADC Mapping	Signal decrease on DWI and increased ADC might indicate treatment response	No specific post-SBRT validation to date; potential area for further research

Our review highlights that changes in T2 signal patterns and reductions in perfusion parameters such as K_trans_ and V_p_ correlate with positive outcomes, including pain relief and local tumor control. These MRI biomarkers help differentiate true response, pseudo-progression, and disease progression, supporting improved patient care. Further multi-institutional validation is needed. Integrating radiomics and machine learning with MRI data enhances response assessment beyond current guidelines. Our study showed that combining radiomics with clinical data improves predictive accuracy, supporting personalized patient monitoring and aligning with precision medicine efforts. However, several limitations exist, including reliance on retrospective studies, varied MRI protocols, and the absence of standardized interpretation criteria. Factors like osseous pseudo-progression and technical requirements further complicate evaluation. Limited prospective evidence and uncertainty regarding long-term predictive value restrict clinical implementation. Future research should focus on standardized multi-parametric MRI assessments and radiomics to confirm their role in guiding spine SBRT follow-up.

We are currently conducting a single-center prospective study using multi-parametric MRI to assess treatment response after spine SBRT. In this trial, we have established the following MRI protocol:: All spine MRI examinations were performed at baseline and after radiotherapy using a 3T system (Vida Fit, Siemens Healthineers, Erlangen, Germany). The pre-contrast protocol included a scout localizer, sagittal T2 TSE STIR (DRB), sagittal T1 TSE (DRB), sagittal 3D T2 SPACE isotropic with axial and coronal reconstructions, axial T2 TSE (DRB), and axial diffusion-weighted imaging with b-values up to 800 s/mm^2^. Following intravenous administration of 16 mL gadoteridol (ProHance), dynamic perfusion imaging was acquired with axial T1 VIBE TWIST using water/fat separation (3 mm). This was followed by post-contrast sagittal T1 TSE (DRB), sagittal subtraction T1 TSE (3 mm), axial fat-saturated T1 TSE (DRB, 3 mm), and high-resolution axial fat-saturated T1 VIBE (DRB, 1 mm). Acquisition parameters and imaging planes were kept identical across time points to ensure reproducible assessment of metastatic spine disease.

When MRI does not provide conclusive information for response assessment after spine SBRT, clinicians should consider alternative imaging strategies. PET/CT serves as a valuable adjunct by detecting metabolic activity and distinguishing tumor recurrence from post-treatment changes such as fibrosis or necrosis [27]. Contrast-enhanced CT helps identify osseous progression and assess postoperative hardware, although it offers limited sensitivity for soft tissue or epidural disease. If imaging remains inconclusive and clinical symptoms suggest progression, a biopsy can provide definitive confirmation and guide further management. By integrating imaging findings with clinical assessment and tumor markers, clinicians can make better decisions in these complex scenarios.

This systematic review evaluated current evidence on MRI-based response assessment after spine SBRT, highlighting the need for more comprehensive approaches. Incorporating advanced techniques like DCE-MRI and DWI alongside conventional MRI could significantly improve post-treatment evaluation.

## CRediT authorship contribution statement

Conceptualization: KD, HH; Data curation: KD, MS, AS, HH, KB; Formal analysis: KD, HH, MS; Funding acquisition: Not applicable; Investigation: KD, MS, HH; Methodology: KD, MS, HH; Project administration: KD, HH; Resources: KD, HH, MS, KB; Software: Not applicable; Supervision: KD, HH; Validation: KD, HH, PJ, AS, DA, MG, JH; Visualization: KD, HH, MS; Writing – original draft: KD, MS, HH; Writing – review & editing: KD, HH, DA, PJ, AS, MG, JH, KB.

## Declaration of competing interest

The authors declare that they have no known competing financial interests or personal relationships that could have appeared to influence the work reported in this paper.

## References

[1] Jaipanya P., Chanplakorn P. (2022). Spinal metastasis: narrative reviews of the current evidence and treatment modalities. J Int Med Res.

[2] Vavourakis M. (2024). Comprehensive insights into metastasis-associated spinal cord compression: pathophysiology, diagnosis, treatment, and prognosis: a state-of-the-art systematic review. J Clin Med.

[3] Sangha A., Korol R., Sahgal A. (2013). Stereotactic body radiotherapy for the treatment of spinal metastases: an overview of the university of Toronto, Sunnybrook health sciences Odette cancer centre, technique. J Med Imaging Radiat Sci.

[4] O’Sullivan R., McDermott M., Keys M., O’Sullivan J.A., Faul C. (2020). Imaging response assessment following stereotactic body radiotherapy for solid tumour metastases of the spine: current challenges and future directions. J Med Imaging Radiat Oncol.

[5] Beaton L., Bandula S., Gaze M.N., Sharma R.A. (2019). How rapid advances in imaging are defining the future of precision radiation oncology. Br J Cancer.

[6] Amini B. (2016). Osseous pseudoprogression in vertebral bodies treated with stereotactic radiosurgery: a secondary analysis of prospective phase I/II clinical trials. Am J Neuroradiol.

[7] Jabehdar Maralani P. (2019). Incidence and time of onset of osseous pseudoprogression in patients with metastatic spine disease from renal cell or prostate carcinoma after treatment with stereotactic body radiation therapy. Clin Neurosurg.

[8] Page M.J. (2021). The PRISMA 2020 statement: an updated guideline for reporting systematic reviews. BMJ.

[9] Hwang Y.J. (2012). Radiosurgery for metastatic spinal tumors: follow-up MR findings. Am J Neuroradiol.

[10] Spratt D.E. (2016). Early magnetic resonance imaging biomarkers to predict local control after high dose stereotactic body radiotherapy for patients with sarcoma spine metastases. Spine J.

[11] Tseng C.L. (2018). Imaging-based outcomes for 24 Gy in 2 daily fractions for patients with de novo spinal metastases treated with spine stereotactic body radiation therapy (SBRT). Int J Radiat Oncol Biol Phys.

[12] Zeng K.L. (2019). Stereotactic body radiotherapy for spinal metastases at the extreme ends of the spine: imaging-based outcomes for cervical and sacral metastases. Clin Neurosurg.

[13] Chen Y., Zhang E., Wang Q., Yuan H., Zhuang H., Lang N. (2021). Use of dynamic contrast-enhanced MRI for the early assessment of outcome of CyberKnife stereotactic radiosurgery for patients with spinal metastases. Clin Radiol.

[14] Wong E., Howard P., Chan A.K.M., Atenafu E.G., Lu H., Tyrrell P. (2021). The initial step towards establishing a quantitative. Magnet Resonance Imaging-Based Framework.

[15] Sahgal A. (2021). Articles Stereotactic body radiotherapy versus conventional external beam radiotherapy in patients with painful spinal metastases: an open-label, multicentre, randomised , controlled , phase 2 / 3 t. Lancet Oncol.

[16] Correia D. (2022). Response assessment after stereotactic body radiation therapy for spine and non-spine bone metastases: results from a single institutional study. Radiat Oncol.

[17] Vellayappan B. (2022). Quantifying the changes in the tumour vascular micro-environment in spinal metastases treated with stereotactic body radiotherapy – a single arm prospective study. Radiol Oncol.

[18] Chen Y. (2023). MRI feature-based radiomics models to predict treatment outcome after stereotactic body radiotherapy for spinal metastases. Insights Imaging.

[19] Chen Y. (2023). Predictive model based on DCE-MRI and clinical features for the evaluation of pain response after stereotactic body radiotherapy in patients with spinal metastases. Eur Radiol.

[20] Chen H. (2024). Magnetic resonance imaging frequency after stereotactic body radiation therapy for spine metastases. Int J Radiat Oncol Biol Phys.

[21] Burgess L. (2024). Practice and principles of stereotactic body radiation therapy for spine and non-spine bone metastases. Clin Transl Radiat Oncol.

[22] Thibault I. (2015). Response assessment after stereotactic body radiotherapy for spinal metastasis: a report from the SPIne response assessment in neuro-oncology (SPINO) group. Lancet Oncol.

[23] Jabehdar Maralani P. (2022). Proposing a quantitative MRI-based linear measurement framework for response assessment following stereotactic body radiation therapy in patients with spinal metastasis. J Neurooncol.

[24] Lee K.R. (2016). Interpretation of MR imaging of spinal metastasis: focus on the understanding of its pathophysiology and the next step toward a further clinical approach using MRI findings. Investig Magn Reson Imaging.

[25] Mulyadi R., Putri P.P., Handoko R.A., Zairinal, Prihartono J. (2023). Dynamic contrast-enhanced magnetic resonance imaging parameter changes as an early biomarker of tumor responses following radiation therapy in patients with spinal metastases: a systematic review. Radiat Oncol J.

[26] Kumar K.A. (2017). A pilot study evaluating the use of dynamic contrast-enhanced perfusion MRI to predict local recurrence after radiosurgery on spinal metastases. Technol Cancer Res Treat.

[27] Azadbakht J, et al., The role of CT and MR imaging in stereotactic body radiotherapy of the spine: from patient selection and treatment planning to post-treatment monitoring; 2024. 10.3390/cancers16213692 PMC11545634 39518130

[28] Byun W.M., Shin S.O., Chang Y., Lee S.J., Finsterbusch J., Frahm J. (2002). Diffusion-weighted MR imaging of metastatic disease of the spine: assessment of response to therapy. Am J Neuroradiol.

[29] Bahig H. (2016). A study of pseudoprogression after spine stereotactic body radiation therapy. Int J Radiat Oncol Biol Phys.

[30] Ruzevick J., Kleinberg L., Rigamonti D. (2014). Imaging changes following stereotactic radiosurgery for metastatic intracranial tumors: Differentiating pseudoprogression from tumor progression and its effect on clinical practice. Neurosurg Rev.

[31] Park S., Do Huh J. (2023). Bone metastases with post-treatment intralesional fatty content of the spine: imaging features from T1-weighted imaging with CT finding correlations. Acta Radiol.

[32] Burgess L. (2022). Predictors of vertebral compression fracture following spine stereotactic body radiotherapy vary with cause: iatrogenic or in conjunction with local failure. Int J Radiat Oncol Biol Phys.

[33] Mossa-Basha M. (2019). Spinal metastasis: diagnosis, management and followup. Br J Radiol.

[34] Arevalo-Perez J., Peck K.K., Lyo J.K., Holodny A.I., Lis E., Karimi S. (2015). Differentiating benign from malignant vertebral fractures using T1-weighted dynamic contrast-enhanced MRI. J Magn Reson Imaging.

[35] Geith T. (2013). Quantitative analysis of acute benign and malignant vertebral body fractures using dynamic contrast-enhanced MRI. Am J Roentgenol.

[36] Guninski R.S. (2024). Efficacy and safety of SBRT for spine metastases: a systematic review and meta-analysis for preparation of an ESTRO practice guideline. Radiother Oncol.

[37] Guckenberger M. (2024). ESTRO clinical practice guideline: Stereotactic body radiotherapy for spine metastases. Radiother Oncol.

